# Management of Labral Tears in the Hip: A Consensus Statement

**DOI:** 10.1177/23259671241305409

**Published:** 2025-01-23

**Authors:** Bogdan A. Matache, Étienne L. Belzile, Olufemi R. Ayeni, Luc De Garie, Ryan M. Degen, Richard Goudie, Martin Heroux, Marie-Josee Klett, Erika Persson, Ivan Wong, The AAC-CASEM Consensus Group, Firas Al-Rawi, Penny-Jane Baylis, Paul E. Beaule, Richard Blanchet, Jordan Buchko, Pierre Collin, Jason Crookham, Bobby Homayoon, Eoghan T. Hurley, Kelly Johnston, Moin Khan, Diane Lambert, Claire Leblanc, Devin Lemmex, Patrick Ling, Parth Lodhia, Billy Longland, R. Kyle Martin, Mark McConkey, Bob McCormack, Mickey Moroz, Marie-Lyne Nault, Ross Outerbridge, Julie Peltz, Anita Pozgay, David Reid, Scott Shallow, Ryan Shields, Allison Tucker, Nathan Urquhart, Jarret Woodmass

**Affiliations:** The Ottawa Hospital, Ottawa, Ontario, Canada; The University of Ottawa, Ottawa, Ontario, Canada; CHU de Quebec-Universite Laval, Quebec, Canada; McMaster University, Hamilton, Ontario, Canada; Universite de Montreal, Montreal, Quebec, Canada; Fowler Kennedy Sports Medicine, London, Ontario, Canada; Western University, London, Ontario, Canada; STRIVE Sport & Exercise Medicine Clinic, Barrie, Ontario, Canada; University of Saskatchewan, Regina, Saskatchewan, Canada; The University of Ottawa, Ottawa, Ontario, Canada; Glen Sather Sports Medicine Clinic, Edmonton, Alberta, Canada; University of Alberta, Edmonton, Alberta, Canada; Dalhousie University, Halifax, Nova Scotia, Canada; University of Toronto, Toronto, Ontario, Canada; McGill University, Montreal, Quebec, Canada; The Ottawa Hospital and The University of Ottawa, Ontario, Canada; Clinique Medicale Pierre-Bertrand, Quebec, Canada; University of Saskatchewan, Regina, Saskatchewan, Canada; Clinique du PEPS-Universite Laval, Quebec, Canada; Pacific Orthopedics and Sports Medicine, Vancouver, British Columbia, Canada; University of Calgary, Calgary, Alberta, Canada; Duke University, Durham, North Carolina, USA; University of Calgary, Calgary, Alberta, Canada; McMaster University, Hamilton, Ontario, Canada; Clinique du PEPS-Universite Laval, Quebec, Canada; McGill University, Montreal, Quebec, Canada; University of Calgary, Calgary, Alberta, Canada; Avant Sports Medicine, Saskatoon, Saskatchewan, Canada; University of British Columbia, Vancouver, British Columbia, Canada; University of British Columbia, Vancouver, British Columbia, Canada; University of Minnesota, Saint Cloud, Minnesota, USA; University of British Columbia, Vancouver, British Columbia, Canada; University of British Columbia, Vancouver, British Columbia, Canada; McGill University, Montreal, Quebec, Canada; Universite de Montreal, Montreal, Quebec, Canada; Kamloops Orthopedics, Kamloops, British Columbia, Canada; Algonquin College, Ottawa, Ontario, Canada; The University of Ottawa, Ottawa, Ontario, Canada; Glen Sather Sports Medicine Clinic and University of Alberta, Edmonton, Alberta, Canada; Queen’s University, Kingston, Ontario, Canada; University of Calgary, Calgary, Alberta, USA; Dalhousie University, Halifax, Nova Scotia, Canada; Dalhousie University, Halifax, Nova Scotia, Canada; Pan Am Clinic and University of Manitoba, Winnipeg, Manitoba, Canada; Investigation performed at The Ottawa Hospital, Ottawa, Ontario, Canada

**Keywords:** femoroacetabular impingement, hip, hip arthroscopy, labral repair, labral tears, nonoperative management

## Abstract

**Background:**

Inconsistencies in the workup of labral tears in the hip have been shown to result in a delay in treatment and an increased cost to the medical system.

**Purpose:**

To establish consensus statements among Canadian nonoperative/operative sports medicine physicians via a modified Delphi process on the diagnosis, nonoperative and operative management, and rehabilitation and return to play (RTP) of those with labral tears in the hip.

**Study Design:**

A consensus statement.

**Methods:**

A total of 40 sports medicine physicians (50% orthopaedic surgeons) were selected for participation based on their level of expertise in the field. Experts were assigned to 1 of 4 balanced working groups defined by specific subtopics of interest. Consensus, strong consensus, and unanimous consensus were defined as achieving 80% to 89%, 90% to 99%, and 100% agreement with a proposed statement, respectively.

**Results:**

There was a unanimous consensus that several prognostic factors—including age, pain severity, dysplasia, and degenerative changes—should be taken into consideration with regard to the likelihood of surgical success. There was strong agreement that the cluster of symptoms of anterior groin pain, pain in hyperflexion, and sharp catching pain with rotation make a diagnosis of a labral tear more likely, that radiographs—including a minimum of a standing anteroposterior pelvis and 45° Dunn view—should be obtained in all patients presenting with a suspected labral tear, that a diagnostic injection should be performed if there is uncertainty that the pain is intra-articular in origin, and that a minimum of 6 months should elapse after surgical treatment before reinvestigation for persistent symptoms.

**Conclusion:**

Overall, 76% of statements reached a unanimous/strong consensus, thus indicating a high level of agreement between nonoperative sports medicine physicians and orthopaedic surgeons on the management of labral tears in the hip. The statements that achieved unanimous consensus included the timing of RTP after surgery, prognostic factors affecting surgical success, and the timing to begin sport-specific training after nonoperative management. There was no consensus on the use of orthobiologics for nonoperative management, indications for bilateral surgery, whether the postoperative range of motion and weightbearing restrictions should be employed, and whether postoperative hip brace usage is required.

Labral tears in the hip and femoroacetabular impingement (FAI) are common sources of nonarthritic hip pain.^7^ Treating these lesions depends on the appropriate diagnosis, initial workup, and referral for surgical management when indicated. Given the differences in practice patterns that exist between the various types of medical practitioners treating this pathology, it is unclear whether management is standardized between nonoperative sports medicine physicians and orthopaedic surgeons.^14,20,24^ Inconsistencies in the workup of these patients have been shown to result in a delay in treatment and an increased cost to the medical system.^12^

Because of the lack of high-quality literature on the subject, consensus statements generated by agreement between experts in the field from both the nonoperative and operative ends of the spectrum are an important source of evidence to help guide the treatment of patients with labral tears in the hip. Furthermore, the definition of a regionally standardized set of guidelines on the diagnosis, nonoperative and operative management, and rehabilitation and return to play (RTP) of labral tears in the hip would help reduce extraneous diagnostic testing and expedite appropriate care of these patients to improve outcomes.

The Arthroscopy Association of Canada-Canadian Academy of Sport and Exercise Medicine (AAC-CASEM) Consensus Group was created with a mandate to establish clinical guidelines for key aspects of the treatment of labral tears in the hip—including diagnosis, nonoperative and operative management, and rehabilitation and RTP. This study aimed to establish consensus statements among nonoperative sports medicine physicians and orthopaedic surgeons with a hip arthroscopy practice via a modified Delphi process on the diagnosis, nonoperative and operative management, and rehabilitation and RTP of labral tears in the hip.

## Methods

### Consensus Working Groups

A total of 40 sports medicine physicians with expertise in the management of FAI, of whom 20 were orthopaedic surgeons with a hip arthroscopy practice (AAC) and 20 were nonoperative physicians (CASEM) participated in generating consensus statements on labral tears in the hip. Experts were assigned to 1 of 4 working groups defined by specific subtopics of interest, as follows: (1) Diagnosis; (2) Nonoperative Management; (3) Operative Management; and (4) Rehabilitation and RTP. AAC and CASEM participants were evenly distributed into working groups to ensure these were balanced in terms of scope of practice to limit bias in opinion. Thus, each working group was randomly assigned 5 AAC and 5 CASEM participants. A liaison (E.T.H.) served as the primary point of contact and facilitated communication and the distribution of surveys to ensure consistency across the working groups. To reduce the potential for bias in the data analysis and/or literature review, the liaison did not submit answers to the questionnaires or participate in the voting process.

### Delphi Consensus Method

Four working groups covering the principal topics of interest in the area of labral tears in the hip were established. A set of questions pertaining to each working group was generated based on clinical relevance and controversy. The Delphi method was used to generate consensus statements for each working group, with groups completing 3 initial rounds of questionnaires, followed by amendments, and lastly a final vote. Questions progressed from an open-ended to a more structured format and were designed to elucidate areas of agreement and disagreement between group members. Once a preliminary consensus statement was generated within a working group, participants were asked whether they “strongly disagreed,”“disagreed,”“neutral,”“agreed,” or “strongly agreed” with it. If there was unanimous agreement within a group on a preliminary consensus statement, this statement was elevated to a final vote. If the agreement was not unanimous within a group, these questions were subject to further discussion by members of that group. The final voting process allowed all study participants to assess the consensus statements generated by the other working groups and vote on whether they “strongly disagreed,”“disagreed,”“neutral,”“agreed,” or “strongly agreed” with them. Surveys were distributed in a blinded fashion using RedCap (Vanderbilt University).

### Final Voting

After the final votes for each question, the degree of agreement was expressed using a percentage of “agreement” or “strong agreement” responses rounded to the nearest whole number. Consensus was defined as 80% to 89%, whereas strong consensus was defined as 90% to 99%, and unanimous consensus was indicated by receiving 100% of the votes in favor of a proposed statement.

## Results

### Diagnosis

Of the 9 total questions and consensus statements in this group, 7 achieved strong consensus and 2 achieved consensus (Table 1).

**Table 1:** Diagnosis^*a*^

Questions and Answers	Strong Disagreement	Disagreement	Neutral	Agreement	Strong Agreement	Consensus
Q1: What are the risk factors and/or mechanism of injury for sustaining a labral tear?	3	0	0	38	60	Strong
A: The risk factors and/or mechanisms of injury for sustaining a labral tear are as follows: (1) cam lesion; (2) pincer lesion; (3) dysplasia; (4) trauma; (5) overuse or misuse; (6) connective tissue disease; and (7) sports with repetitive or sustained flexion/adduction/internal rotation.						
Q2: What are the clusters of symptoms in patient history that have a higher likelihood of indicating a labral tear? Are there any differences in presentation between pediatric and adult patients?	5	0	3	43	49	Strong
A: The cluster of symptoms on patient history that have a higher likelihood of indicating a labral tear is as follows: (1) anterior groin pain; (2) pain in hyperflexion or sustained flexion; and (3) sharp, catching pain with rotation. Pediatric and adult patients have similar symptoms on presentation.						
Q3: What physical examination maneuvers should be used to diagnose a labral tear?	0	5	8	54	32	Consensus
A: The physical examination maneuvers that should be used to diagnose a labral tear include (1) FADIR, (2) FABER, and (3) ROM.						
Q4: Should radiographs be obtained in all patients presenting with a suspected labral tear? If so, for what reasons? Which views?	0	3	3	41	54	Strong
A: Yes, radiographs should be obtained in all patients presenting with a suspected labral tear, including a minimum of (1) standing AP pelvis and (2) 45° Dunn view.						
Q5: How should a clinically significant labral tear be graded/classified?	0	3	8	62	27	Consensus
A: A clinically significant labral tear should be graded/classified by (1) pain/no pain, (2) stable/unstable, (3) repairable/unrepairable, (4) underlying bony morphology, and (5) underlying osteoarthritis.						
Q6: Which advanced imaging modality is preferred for a patient presenting with a suspected/known labral tear: CT, MRI, or MRA?	0	3	3	51	43	Strong
A: MRA is preferred for a patient presenting with a suspected/known labral tear.						
Q7: When should a diagnostic hip injection (“Xylotest”) be performed in a patient presenting with a suspected/known labral tear? Should a corticosteroid be used in addition to the local anesthetic?	0	0	3	46	51	Strong
A: A diagnostic hip injection should be performed in a patient presenting with a suspected/known labral tear if there is diagnostic uncertainty of whether the pain is coming from the hip joint. Corticosteroids can be used if there is a degenerative etiology underlying it.						
Q8: What is the differential diagnosis for a patient presenting with a suspected/known labral tear?	0	0	8	43	49	Strong
A: The differential diagnosis should include (1) FAI, (2) osteoarthritis, (3) chondral injury, (4) dysplasia, (5) referred back pain, (6) tendinitis, (7) impingement, and (8) snapping hip.						
Q9: When should reinvestigation for a patient who has already received surgical treatment for a labral tear be considered?	0	0	5	51	43	Strong
A: After a minimum of 6 months, reinvestigation for a patient who has already received surgical treatment for a labral tear should be considered if they have (1) new pain, (2) new loss of motion, (3) new injury, and (4) persistent symptoms postoperatively.						

a Data are presented as %. A, answer; AP, anteroposterior; CT, computed tomography; FABER, flexion, abduction and external rotation; FADIR, flexion, adduction, and internal rotation; FAI, femoroacetabular impingement; MRA, magnetic resonance arthrography; MRI, magnetic resonance imaging; Q, question; RTP, return to play; ROM, range of motion.

### Nonoperative Management

Of the 9 total questions and consensus statements in this group, 2 achieved unanimous consensus, 5 achieved strong consensus, 1 achieved consensus, and 1 failed to reach consensus (Table 2).

**Table 2:** Nonoperative Management^*a*^

Questions and Answers	Strong Disagreement	Disagreement	Neutral	Agreement	Strong Agreement	Consensus
Q1: What are the indications for nonoperative management of labral tears?	0	0	0	30	70	Unanimous
A: It is reasonable to trial nonoperative management in almost all patients. The relative indications for nonoperative management of labral tears include (1) minimal pain, (2) chronic tear, (3) small tear, (4) older athlete, (5) recreational athlete, (6) no mechanical symptoms, and (7) no limitations in activities of daily living.						
Q2: What are the contraindications for nonoperative management of labral tears?	0	0	8	70	22	Strong
A: The relative contraindications for nonoperative management of labral tears include those with significant mechanical symptoms.						
Q3: Does patient age play a role in the indications/contraindications for nonoperative management of a labral tear? If so, how?	0	0	3	43	54	Strong
A: Age plays a relative role as younger age may be an indicator for more aggressive treatment of a labral tear, and older age may be a surrogate for osteoarthritis or a more degenerative etiology.						
Q4: Should the conservative management of labral tears be conducted in a multidisciplinary fashion? If so, who should comprise the multidisciplinary team?	0	0	0	27	73	Strong
A: Yes, conservative management of labral tears should be conducted in a multidisciplinary fashion and be composed of surgeons, physicians, physical therapists, and trainers.						
Q5: What are the prognostic factors that should be taken into consideration with regard to the likelihood of success in patients undergoing nonoperative management of labral tears?	0	0	3	35	62	Strong
A: The prognostic factors that should be taken into consideration with regard to the likelihood of success in patients undergoing nonoperative management of labral tears include (1) age, (2) severity of pain, (3) mental health, (4) comorbidities, (5) obesity, (6) failed prior rehabilitation, (7) duration of symptoms, (8) pincer lesion, (9) cam lesion, (10) joint degeneration, (11) dysplasia, (12) motivation, and (13) workers’ compensation.						
Q6: What are the key components of nonoperative management of labral tears?	0	6	3	27	70	Strong
A: The key components of nonoperative management of labral tears include (1) ROM, (2) strength, (3) core strengthening, (4) gluteal stabilization, (5) functional movement retraining, (6) symptom management, (7) activity modification, and (8) patient education.						
Q7: When are patients allowed to begin sport-specific training when being treated nonoperatively for a labral tear? Does this vary depending on the sport?	0	0	0	27	73	Unanimous
A: There is no specific time point to begin sport-specific training when being treated nonoperatively for a labral tear; it is dependent on (1) strength, (2) pain, and (3) apprehension. It varies depending on the sport and the ability to complete sport-specific symptoms.						
Q8: Is there a role for corticosteroid injections in the nonoperative management of labral tears? Are there any contraindications for the use of corticosteroid injections in the nonoperative management of labral tears?	0	14	5	49	32	Consensus
A: There is a role for corticosteroid injections in the nonoperative management of labral tears for diagnostic purposes and symptom management. There are no contraindications for the use of corticosteroid injections in the nonoperative management of labral tears.						
Q9: Is there a role for orthobiologics in the nonoperative management of labral tears? If so, which ones and at which frequency? Please be specific.	0	3	22	64	11	None
A: There is a role for PRP and viscosupplementation in the nonoperative management of labral tears.						

a Data are provided as %. A, answer; PRP, platelet-rich plasma; Q, question; ROM, range of motion.

### Operative Management

Of the 12 total questions and consensus statements in this group, 1 achieved unanimous consensus, 8 achieved strong consensus, 1 achieved consensus, and 2 failed to reach consensus (Table 3).

**Table 3:** Operative Management^*a*^

Questions and Answers	Strong Disagreement	Disagreement	Neutral	Agreement	Strong Agreement	Consensus
Q1: What are the indications for operative management of labral tears (labral repair, reconstruction, or debridement ± osteochondroplasty)?	0	0	3	38	59	Strong
A: The relative indications for operative management of labral tears include (1) young or competitive athlete, (2) significant mechanical symptoms, (3) limitations in activities of daily living, and (4) moderate-severe pain.						
Q2: What are the contraindications for operative management of labral tears (labral repair, reconstruction, or debridement ± osteochondroplasty)?	0	0	5	41	54	Strong
A: The relative contraindications for operative management of labral tears include (1) minimal pain, and (2) minimal limitations in activities of daily living.						
Q3: What are the prognostic factors that should be taken into consideration with regard to the likelihood of success in patients undergoing operative management of labral tears?	0	0	0	46	54	Unanimous
A: The prognostic factors that should be taken into consideration with regard to likelihood of success in patients undergoing operative management of labral tears include (1) age, (2) severity of pain, (3) mental health, (4) comorbidities, (5) obesity, (6) failed prior rehabilitation, (7) duration of symptoms, (8) pincer lesion, (9) cam lesion, (10) joint degeneration, (11) dysplasia, (12) motivation, and (13) workers’ compensation.						
Q4: What is the acceptable delay between the time of diagnosis by a nonoperative physician and the time of initial consultation with an orthopaedic surgeon?	0	5	8	54	32	Consensus
A: Delays in consultation with an orthopaedic surgeon after diagnosis by a nonoperative physician should not exceed 3 to 6 months.						
Q5: What are the potential negative impacts of delaying operative management of labral tears?	0	0	3	48	49	Strong
A: The potential negative impacts of delaying operative management of labral tears include (1) persistent pain, (2) increasing labral tear size/complexity, (3) increasing chondral damage, (4) delayed RTP/function, (5) worse deconditioning, (6) decline in mental health, and (7) missed work/school.						
Q6: What are the indications for performing bilateral surgery in patients undergoing operative management of labral tears?	0	8	14	59	19	None
A: The indication for performing bilateral surgery in patients undergoing operative management of labral tears is bilateral symptomatic disease with both having indications for symptomatic disease.						
Q7: What are the contraindications for performing bilateral surgery in patients undergoing operative management of labral tears?	0	0	5	60	35	Strong
A: The contraindications for performing bilateral surgery in patients undergoing operative management of labral tears are (1) poor postoperative support and (2) poor ability to comply with weightbearing restrictions.						
Q8: Is there a preferred surgical approach (open vs arthroscopic) for labral repair, reconstruction, or debridement ± osteochondroplasty?	0	0	8	31	61	Strong
A: Arthroscopic surgery is the preferred surgical approach over open surgery for labral repair, reconstruction, or debridement ± osteochondroplasty.						
Q9: What are the potential complications of operative management of labral tears that patients should be informed of?	0	0	3	53	44	Strong
A: The potential complications of operative management of labral tears that patients should be informed of include (1) no improvement or worsening of symptoms, (2) ongoing pain, (3) potential for reinjury, (4) loss of ROM, (5) DVT, (6) infection, (7) nerve damage (pudendal, femoral, or LFCN), and (8) heterotopic ossification.						
Q10: What are the indications for revision surgery in patients who have ongoing symptoms after operative management of labral tears?	0	0	8	61	31	Strong
A: The relative indications for revision surgery in patients who have ongoing symptoms after operative management of labral tears include (1) >12 months of pain postoperatively, (2) imaging-confirmed pathology, (3) completed appropriate postoperative rehab, (4) cam underresection, (5) hip instability requiring capsular reconstruction, and (6) positive response to diagnostic hip injection.						
Q11: What are the contraindications for revision surgery in patients who have ongoing symptoms after operative management of labral tears?	0	0	6	47	47	Strong
A: The relative contraindications for revision surgery in patients who have ongoing symptoms after operative management of labral tears include (1) failure to comply with postoperative physical therapy, (2) symptom duration <6 months, (3) poor response to diagnostic hip injection, and (4) arthritic development/progression.						
Q12: Should patients be placed in a hip abduction brace postoperatively? If so, for how long? And when should bracing begin?	3	0	25	33	39	None
A: Patients should not be placed in a hip abduction brace postoperatively.						

a Data are presented as %. A, answer; DVD, dissociated vertical deviation; LFCN, lateral femoral cutaneous nerve; Q, question; ROM, range of motion; rehab, rehabilitation; RTP, return to play.

### Rehabilitation and RTP

Of the 11 total questions and consensus statements in this group, 1 achieved unanimous consensus, 7 achieved strong consensus, 1 achieved consensus, and 2 failed to reach consensus (Table 4).

**Table 4:** Rehabilitation and RTP^*a*^

Questions and Answers	Strong Disagreement	Disagreement	Neutral	Agreement	Strong Agreement	Consensus
Q1: Should there be any hip ROM restrictions after surgery? If so, which ones and for how long?	0	3	22	56	19	None
A: Hip ROM restrictions after surgery should be based on the extent of the surgery performed.						
Q2: Should there be any weightbearing restrictions after surgery? If so, for how long?	3	6	31	46	14	None
A: Hip weightbearing after surgery should be limited to partial weightbearing for 2 weeks.						
Q3: How long after surgery may patients resume an isometric strengthening program?	0	0	8	61	31	Strong
A: Patients may resume an isometric strengthening program within the first week.						
Q4: How long after surgery may patients resume an eccentric strengthening program?	0	0	3	75	22	Strong
A: Patients may resume an eccentric strengthening program within 4 to 6 weeks.						
Q5: How long after surgery may patients resume a concentric strengthening program?	0	0	8	70	22	Strong
A: Patients may resume a concentric strengthening program within 4 to 6 weeks.						
Q6: What criteria should be considered for RTP after operative management of labral tears?	0	3	0	36	61	Strong
A: The criteria that should be considered for RTP after operative management of labral tears include (1) pain-free, (2) full ROM, (3) >90% of contralateral strength in flexion, abduction, adduction, and core strength, (4) sport-specific endurance, (5) psychological readiness, (6) balance, and (7) proprioception.						
Q7: Is there a minimum amount of time from surgery to RTP after operative management of labral tears? Does the type of procedure performed affect this duration?	0	0	8	56	36	Strong
A: The minimum amount of time from surgery to RTP after operative management of labral tears is 3 months for a labral debridement and 4-6 months for labral repairs.						
Q8: What aspects of physical examination should be included when determining when to allow patients to RTP?	0	3	3	46	48	Strong
A: It should be based on sport-specific skills.						
Q9: Does the type of sport played affect the timing of RTP? If so, how?	0	0	0	51	49	Strong
A: Cutting sports and deep flexion sports take longer to RTP.						
Q10: Is the timing of RTP affected by whether a unilateral or bilateral procedure was performed?	0	0	0	49	51	Unanimous
A: Yes, RTP may take longer if a bilateral procedure is performed.						
Q11: Should sports psychology testing be included when determining when to allow players to RTP?	0	2	14	46	38	Consensus
A: Sports psychology testing should be included if available when determining when to allow players to RTP.						

a Data are presented as %. A, answer; Q, question; ROM, range of motion; RTP, return to play.

## Discussion

The principal findings of this study are that a high level of agreement exists between nonoperative sports medicine physicians and orthopaedic surgeons with regard to the management of labral tears in the hip. Specifically, the agreement is highest on the diagnosis and nonoperative management of labral tears, while significant disagreement exists on the role of orthobiologics, indications for bilateral surgery, use of hip abduction bracing postoperatively, and range of motion (ROM) and weightbearing restrictions after surgery.

Diagnosis of a labral tear in the hip relies on appropriate identification of the risk factors and clinical presentation of patients with this pathology. Kahlenberg et al^12^ showed that patients with FAI saw a mean of 4 health care providers, had a mean of 3 diagnostic tests, and tried a mean of 3 treatments before appropriate diagnosis, resulting in a mean US$1800 higher health care dollars spent above the minimum cost for each patient. There was a strong consensus in our study that the risk factors for sustaining a labral tear in the hip include the presence of a cam or pincer lesion, acetabular dysplasia, trauma, and overuse or misuse (such as from sports with repetitive or sustained flexion/adduction/internal rotation). In a magnetic resonance imaging (MRI) study of patients with FAI, Kassarjian et al^13^ showed that 100% of patients with clinically symptomatic cam lesions had an associated anterosuperior labral tear. Labral tears also have a high prevalence in patients with hip dysplasia, owing to the abnormal hypertrophy and loading of the labrum.^27^ Moreover, Epstein et al^5^ showed that labral tears accounted for 69.1% of all intra-articular hip pathologies in professional ice hockey players, resulting in a mean of 8 man-games missed per injury. There was also a strong consensus that patients with a labral tear tend to present with a cluster of symptoms that includes anterior groin pain, pain in hyperflexion or sustained flexion, and sharp/catching pain with rotation and that the presentation is similar in pediatric and adult patients. This latter point is supported by a study by Sink et al,^26^ where anterior groin pain and pain/functional limitations in flexion were the most commonly encountered symptoms in an adolescent population.

Diagnostic imaging in the workup of labral tears is an area of controversy, with a general lack of guidelines available pertaining to the indications for plain radiography, computed tomography (CT), MRI, and magnetic resonance arthrography (MRA). There was a strong consensus that radiographs should be obtained for all patients with a suspected labral tear—including a minimum of a standing anteroposterior pelvis and a 45° Dunn view. In addition, there was a strong consensus that MRA is the preferred advanced imaging modality in this patient population. This is somewhat in discordance with a systematic review by Reiman et al^23^ that showed CT arthrography as superior to MRA in diagnosing labral tears. They also demonstrated that despite this, these advanced imaging modalities have somewhat of a limited clinical utility given the high degree of pretest probability for accurately diagnosing labral tears based on clinical presentation and plain radiography alone. Barton et al^2^ compared the accuracy of plain radiography in the diagnosis of cam-type FAI to MRI and showed that the Dunn view had a high sensitivity, specificity, positive and negative predictive value, and accuracy (91%, 88%, 93%, 84%, and 90%, respectively) for diagnosing cam-type FAI, as well as a high correlation (*r* = 0.702) to MRI. These findings support the practice patterns of some of the study participants who choose to forego advanced imaging when the clinical presentation and plain radiographic findings are typical for a hip labral tear.

The role of injections in the management of labral tears in the hip is also an area of controversy. Our study showed strong agreement that a diagnostic hip injection should be performed in a patient presenting with a suspected/known labral tear if there is diagnostic uncertainty of whether the pain is coming from the hip joint and that corticosteroids can be used if there is a degenerative cause underlying it. This is supported by a previous study by Chinzei et al^4^ who found significantly improved 1-year postoperative outcomes in patients who had a >50% pain relief response after preoperative diagnostic injection. Similarly, Gao et al^8^ evaluated 78 patients with atypical symptoms and showed that a positive response to a preoperative diagnostic injection was 91.7% accurate for detecting intra-articular pathology. In addition, Krych et al^15^ demonstrated that the addition of corticosteroids provided limited therapeutic benefit in patients with FAI who did not have degenerative changes, thus reinforcing the notion that corticosteroids should be reserved for patients with Tonnis ≥2 changes. With regard to orthobiologics, our study failed to reach an agreement on the role of these therapeutic injections in the management of labral tears. This is in keeping with the low level of evidence surrounding this subject, highlighting this as an area in need of further study.^21^

A strong consensus was reached about the prognostic factors to be taken into consideration with regard to the likelihood of success after operative management of labral tears, including patient age. Bryan et al^3^ showed that age >55 years was associated with a higher incidence of full-thickness cartilage defects (22% vs 4%) and requirement for labral debridement instead of repair (78% vs 36%), as well as less significant improvements in functional scores at 2 years as compared with the younger age cohort in their study of 201 patients with FAI. Similarly, Shanmugaraj et al^25^ found that 3 of 17 studies included in their systematic review showed significantly worse outcomes and 2 of 17 studies had a significantly greater rate of conversion to arthroplasty in older compared with younger patients. Despite these findings, older patients in these studies still demonstrated significantly improved functional outcomes from baseline after hip arthroscopy.^17^ These results both support the use of this treatment option in this population while also highlighting the importance of appropriate patient selection. Mental health status was also identified as an important prognostic factor. In their study of 64 patients who underwent surgical treatment of FAI, Jacobs et al^11^ showed that symptom severity had a significantly higher correlation with mental health status than the labral tear size of FAI deformity. Similarly, in a larger study, Lynch et al^16^ found that baseline mental health scores were as important predictors as baseline hip functional outcome scores of 1-year clinical outcomes after hip arthroscopy. Consensus was also reached, suggesting that sports psychology testing should be included if available when determining when to allow players to RTP. These findings highlight the importance of assessing and facilitating the treatment of mental health issues when present to improve outcomes in patients with labral tears in the hip.

Previous studies and expert groups have demonstrated that physical therapy has an important role to play in the postoperative management of labral tears in the hip.^9,18,22^ However, the specifics of postoperative rehabilitation are an area of ongoing controversy, further highlighted by our lack of consensus pertaining to ROM and weightbearing restrictions and hip abduction brace usage. Only 60% of participants agreed that weightbearing after surgery should be limited to partial weightbearing for 2 weeks. This disagreement is not surprising given the lack of high-quality literature on the subject. In a comparative cohort study of 133 patients undergoing hip arthroscopy for FAI, Avnieli et al^1^ found no difference in terms of patient outcomes, subjective rates of improvement, satisfaction scores, or willingness to undergo the procedure again between the 3-week nonweightbearing and weightbearing as tolerated groups. However, a recent systematic review reported a lack of sufficient comparative evidence to make specific recommendations about postoperative weightbearing.^10^ With regard to postoperative hip bracing, the overall message is much of the same. In an exploratory randomized controlled trial of nonsurgical treatment of FAI, Eyles et al^6^ showed a mild significant improvement in 33-item international Hip Outcome Tool scores in patients who were braced for 6 weeks compared with those who were not, but the confidence intervals were notably wide. Similarly, Newcomb et al^19^ showed that while peak flexion (5.3°), adduction (2.2°), and internal rotation (5.6°) moments were subtly reduced with brace usage, functional outcomes were no different. At this time, the evidence does not seem to support postoperative weightbearing and ROM restrictions, nor hip brace usage, yet this remains an area of treatment variability and clinical disagreement.

### Limitations

This study has several limitations. First, consensus statements are considered to be level 5—expert-opinion level data, which makes them susceptible to inherent biases in the participant selection process. However, we sought to include an equal number of nonoperative sports medicine physicians and orthopaedic surgeons with a hip arthroscopy practice who have expertise in this area, as evidenced by their clinical and academic achievements on the topic. While participants were individually selected, they were randomly allocated in a 1 to 1 ratio to the 4 study groups by the study liaison who was not involved in the voting process. In addition, participants were unaware of which other authors were in their respective groups. Second, there was no standardized process for generating the study questions, thus rendering them at risk of bias. However, during the voting rounds, all participants had the opportunity to contribute to the manuscript and raise points for discussion in a blinded fashion. Last, there are some limitations with the Delphi process itself, as it may represent filtered-down expert opinion with less individual ownership of ideas, ultimately representing level 5 data.

## Conclusion

Overall, 76% of statements reached a unanimous or strong consensus, thus indicating a high level of agreement between nonoperative sports medicine physicians and orthopaedic surgeons on the management of labral tears in the hip. The statements that achieved strong consensus were the timing of RTP after unilateral versus bilateral surgery, the type of sport played affecting the timing of RTP, prognostic factors affecting surgical success, the timing to begin sport-specific training after nonoperative management, and the indications for and the use of a multidisciplinary approach for nonoperative management of labral tears. There was no consensus on the use of orthobiologics for nonoperative management, indications for bilateral surgery, whether the postoperative ROM and weightbearing restrictions should be employed, and whether postoperative hip brace usage is required.

## Authors

Bogdan A. Matache, MDCM, Dip Sport Med (The Ottawa Hospital, Ottawa, Ontario, Canada; The University of Ottawa, Ottawa, Ontario, Canada); Étienne L. Belzile, MD (CHU de Quebec-Universite Laval, Quebec, Canada); Olufemi R. Ayeni, MD, PhD (McMaster University, Hamilton, Ontario, Canada); Luc De Garie, MD, Dip Sport Med (Universite de Montreal, Montreal, Quebec, Canada); Ryan M. Degen, MD (Fowler Kennedy Sports Medicine, London, Ontario, Canada; Western University, London, Ontario, Canada); Richard Goudie, MD, CCFP (SEM), Dip Sport Med (STRIVE Sport & Exercise Medicine Clinic, Barrie, Ontario, Canada); Martin Heroux, MD, CCFP (EM, SEM), FCFP (University of Saskatchewan, Regina, Saskatchewan, Canada); Marie-Josee Klett, MD, MSc, CCFP (SEM), Dip Sport Med (The University of Ottawa, Ottawa, Ontario, Canada); Erika Persson, MD, Dip Sport Med (Glen Sather Sports Medicine Clinic, Edmonton, Alberta, Canada; University of Alberta, Edmonton, Alberta, Canada); Ivan Wong, MD (Dalhousie University, Halifax, Nova Scotia, Canada); and The AAC-CASEM Consensus Group:

Firas Al-Rawi MD (University of Toronto, Toronto, Ontario, Canada); Penny-Jane Baylis (McGill University, Montreal, Quebec, Canada); Paul E. Beaule MD (The Ottawa Hospital and The University of Ottawa, Ontario, Canada); Richard Blanchet MD (Clinique Medicale Pierre-Bertrand, Quebec, Canada); Jordan Buchko MD (University of Saskatchewan, Regina, Saskatchewan, Canada); Pierre Collin MD (Clinique du PEPS-Universite Laval, Quebec, Canada); Jason Crookham MD (Pacific Orthopedics and Sports Medicine, Vancouver, British Columbia, Canada); Bobby Homayoon MD (University of Calgary, Calgary, Alberta, Canada); Eoghan T. Hurley MD, PhD (Duke University, Durham, North Carolina, USA); Kelly Johnston MD (University of Calgary, Calgary, Alberta, Canada); Moin Khan MD (McMaster University, Hamilton, Ontario, Canada); Diane Lambert MD (Clinique du PEPS-Universite Laval, Quebec, Canada); Claire Leblanc MD (McGill University, Montreal, Quebec, Canada); Devin Lemmex MD (University of Calgary, Calgary, Alberta, Canada); Patrick Ling MD (Avant Sports Medicine, Saskatoon, Saskatchewan, Canada); Parth Lodhia MD (University of British Columbia, Vancouver, British Columbia, Canada); Billy Longland MD (University of British Columbia, Vancouver, British Columbia, Canada); R. Kyle Martin MD (University of Minnesota, Saint Cloud, Minnesota, USA); Mark McConkey MD (University of British Columbia, Vancouver, British Columbia, Canada); Bob McCormack MD (University of British Columbia, Vancouver, British Columbia, Canada); Mickey Moroz MD (McGill University, Montreal, Quebec, Canada); Marie-Lyne Nault MD (Universite de Montreal, Montreal, Quebec, Canada); Ross Outerbridge MD (Kamloops Orthopedics, Kamloops, British Columbia, Canada); Julie Peltz MD (Algonquin College, Ottawa, Ontario, Canada); Anita Pozgay MD (The University of Ottawa, Ottawa, Ontario, Canada); David Reid MD (Glen Sather Sports Medicine Clinic and University of Alberta, Edmonton, Alberta, Canada); Scott Shallow (Queen’s University, Kingston, Ontario, Canada); Ryan Shields MD (University of Calgary, Calgary, Alberta, USA); Allison Tucker MD (Dalhousie University, Halifax, Nova Scotia, Canada); Nathan Urquhart MD (Dalhousie University, Halifax, Nova Scotia, Canada); and Jarret Woodmass MD (Pan Am Clinic and University of Manitoba, Winnipeg, Manitoba, Canada).
